# Development of a non-infectious encapsidated positive control RNA for molecular assays to detect foot-and-mouth disease virus

**DOI:** 10.1016/j.jviromet.2015.04.002

**Published:** 2015-08

**Authors:** Mikidache Madi, Valerie Mioulet, Donald P. King, George P. Lomonossoff, Nicholas P. Montague

**Affiliations:** a The Pirbright Institute, Ash Road, Pirbright GU24 0NF, Surrey, United Kingdom; b Department of Biological Chemistry, John Innes Centre, Colney Lane, Norwich NR4 7UH, United Kingdom

**Keywords:** Cowpea mosaic virus, Positive control, Diagnostics, Foot-and-mouth disease virus, RNA

## Abstract

Positive controls are an important component of the quality-control of molecular tests used for diagnosis of livestock diseases. For high consequence agents such as foot-and-mouth disease virus (FMDV), the positive controls required to monitor template extraction, reverse transcription and amplification steps usually consist of material derived from infectious viruses. Therefore, their production is dependent upon the use of high containment facilities and their deployment carries the risks associated with inactivation of “live” FMDV. This paper describes the development of a novel non-infectious positive control that encodes FMDV RNA sequences that are encapsidated within Cowpea mosaic virus (CPMV) particles. This surrogate RNA has been engineered to contain sequences from the 5′UTR and 3D regions of FMDV targeted by many molecular assays (conventional RT-PCR, real-time RT-PCR and RT-LAMP). These sequences were inserted into a movement-deficient version of CPMV RNA-2 which is rescued from cowpea plants (*Vigna unguiculota*) by inoculation with RNA-1. In order to evaluate the performance of these encapsidated RNAs, nucleic acid prepared from a 10-fold dilution series was tested using a range of molecular assays. Results generated by using the molecular assays confirmed RNA-dependent amplification and the suitability of these particles for use in a range of diagnostic tests. Moreover, these CPMV particles were highly stable for periods of up to 46 days at room temperature and 37 °C. Recombinant CPMV can be used to produce high yields of encapsidated RNAs that can be used as positive and negative controls and standards in molecular assays. This approach provides a surrogate that can be potentially used outside of containment laboratories as an alternative to inactivated infectious virus for molecular diagnostic testing.

## Highlights

• FMDV is highly infectious and can only be handled in high-containment laboratories.
• This study has developed encapsidated control particles containing FMDV RNA.
• The construct contains target sequences for molecular assays used to detect FMDV.
• These control particles were evaluated using routine tests used for FMD diagnosis.
• These particles are non-infectious and temperature-stable.

## 1 Introduction

Early detection is key for the control and eventual eradication of many viral diseases. Real-time Reverse Transcription PCR (RT-qPCR) is widely used as a routine diagnostic tool for livestock diseases such as foot-and-mouth disease (FMD; Hoffmann et al., 2009). Nevertheless, the performance of these molecular protocols can be impacted by human errors and the shelf-life of the enzymes used for template amplification. Therefore, processing a surrogate template as a positive control in parallel with clinical samples is a necessary component of validation to rule out false negative results. These positive controls usually consist of material derived from infectious viruses, which for high-impact livestock diseases such as FMD are dependent upon the use of high containment facilities and associated bio-safety risks. An alternative strategy, utilises internal controls produced via in vitro transcription (Heath et al., 2003; Westcott et al., 2003), and studies have indicated that in vitro transcribed RNA can be stored at −20 °C and undergo 40 cycles of freeze–thawing, or stored at 37 °C for 12 days with no negative influence on the subsequent RT-PCR amplification (Hoffmann et al., 2006). However, without special precautions or buffer conditions, these synthetic RNA internal controls are relatively unstable and can easily be degraded by cellular enzymes. In view of these disadvantages, this paper describes the development of a novel non-infectious encapsidated viral RNA that can be used as a positive control in molecular diagnostic tests.

Cowpea mosaic virus (CPMV) is a plant virus which belongs to the *Comoviridae* family within the new order *Picornavirales*. Its genome consists of two molecules of positive sense RNA that are encapsidated separately in very stable icosahedral virus particles which can survive at 60 °C for an hour across a pH range 4–9 (Montague et al., 2011). While RNA-1 is about 6.0 kb in length, RNA-2 is about 3.5 kb, permitting additional lengths of sequence to be inserted into RNA-2 without abolishing its ability to be replicated and packaged in plants (Gopinath et al., 2000; Monger et al., 2006). CPMV shares similarities in terms of genome organisation and capsid structure with Picornaviruses such as FMD virus (FMDV) and swine vesicular disease virus (SVDV). CPMV is a good candidate to provide recombinant viral particles containing heterologous sequences since it can be purified easily and at high yield from infected plants with up to 1 mg of particles per gram of infected leaf tissue (Montague et al., 2011). The possibility of inserting an untranslated FMDV sequence downstream of CPMV RNA-2 sequence encoding the small (S) protein has been previously demonstrated (King et al., 2007). Although the resulting virus particles served as efficient real-time RT-PCR controls, the approach suffered from two disadvantages: the creation of the control constructs was a multistep process and the particles produced retained their ability to give a productive infection in plants. Though the latter problem could be reduced by physically separating the RNA-2-containing particles in a preparation (King et al., 2007), this is a time-consuming and often incomplete process. This paper describes the design of a construct that produces recombinant CPMV particles that are incapable of causing a productive infection on plants and into which heterologous sequences can be inserted in a single step. The particles are shown to form the basis of a highly effective and safe positive control for the molecular dectection of FMDV.

## 2 Materials and methods

### 2.1 Strategy used to produce movement-deficient Cowpea mosaic virus (CPMV)

**Fig. 1:**
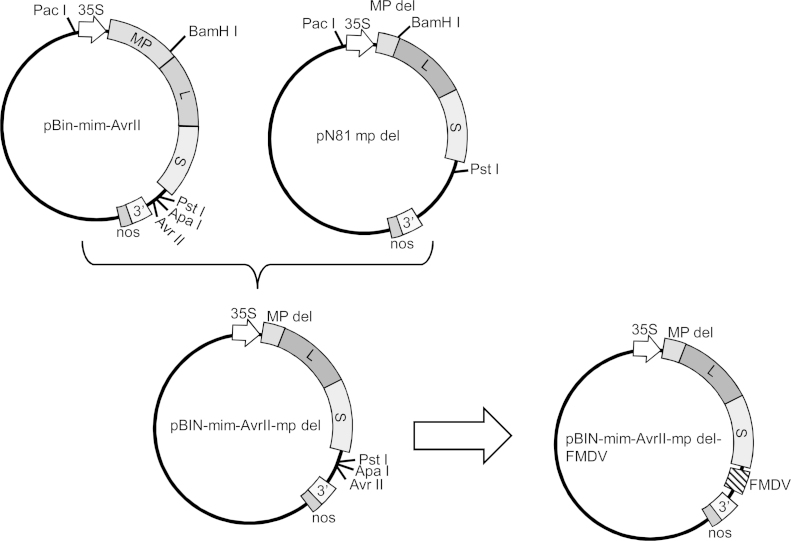
A cartoon outlining the cloning strategy used to create an engineered FMDV surrogate RNA within a movement deficient CPMV particle. The identity of the different plasmids are indicated and the location of Cauliflower mosaic virus 35S promoter (35S), as well as regions encoding CPMV movement protein (MP), partially deleted MP (MP del), large coat protein (L), small coat protein (S), 3′ end of CPMV RNA-2 (3′) and the Agrobacterium nopaline synthase terminator (nos). The location of the artificial construct (FMDV) is shown in pBIN-mim-AvrII-mp del FMDV.

To facilitate one-step cloning of a heterologous sequence into a full-length copy of RNA-2 of CPMV, an ApaI restriction enzyme (RE) digestion site was introduced between the PstI and StuI RE sites in the 3′UTR of plasmid pCVW (Gopinath et al., 2000). The 2 kb BamHI-EcoRI fragment from pCVW ApaI encompassing the 3′ end of RNA-2 was then ligated into a similarly digested fragment from the plasmid pBinP-NS-ER-DsRed (Sainsbury et al., 2008) to create the binary plasmid pBin-mim. An oligonucleotide containing the sequence of an AvrII site was then inserted into the unique ApaI site of pBin-mim to create pBIN-mim-AvrII (Fig. 1). This plasmid allows any sequence with ApaI and AvrII-compatible ends to be cloned downstream of the open reading frame of an infectious clone of CPMV RNA-2 in a single step. To create a movement-deficient version of CPMV, plasmid pN81S2NT, containing a full-length copy of CPMV RNA-2 (Liu and Lomonosoff, 2002) was digested with NdeI and AccI. Following treatment with *Escherichia coli* DNA polymerase I (Klenow fragment) to remove overhangs, the RNA was re-ligated to give plasmid pN81mp del in which a 456 nucleotide section from the 48K movement protein sequence had been removed while retaining the open reading frame of the polyprotein. Restriction enzyme digestion of pN81mp del with PacI and BamHI released a fragment carrying the deleted version of the movement protein; this was substituted for the equivalent wild-type sequence in pBin-mim-AvrII to give plasmid pBIN-mim-AvrII-mp del (Fig. 1). This plasmid allows direct insertion of ApaI-AvrII sequences into a movement-deficient version of RNA-2 (Fig. 1).

### 2.2 Design of artificial construct containing primer sites for FMDV diagnostic assays

**Fig. 2:**
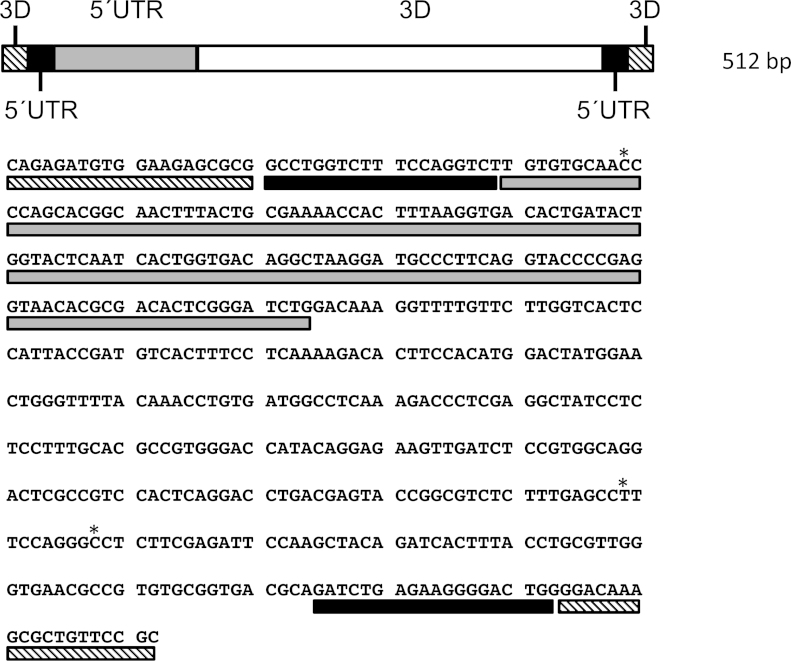
Complete sequence of the engineered FMDV surrogate RNA showing locations of the different regions of the FMDV genome: 3D conventional RT-PCR targets (3D: striped boxes); 5′UTR conventional RT-PCR targets (5′UTR: black boxes); a part of the 5′UTR encompassing a real-time RT-PCR assay (5′UTR: grey boxes) and a part of the 3D encoding region encompassing the molecular targets for a number of diagnostics tests (3D: open box). This sequences is based on the O1-Manisa FMDV reference isolate and incorporates point nucleotide substitutions (*) to reduce the mismatch of the engineered construct with the primer and probe sequences used to detect FMDV.

A 512 bp nucleic acid sequence was designed to contain the molecular targets for two conventional (agarose gel-based) RT-PCRs, two RT-qPCR and two RT-LAMP assays used to detect FMDV (Fig. 2). This fragment embodies forward and reverse primer sites (shown in Table 1), short flanking regions and probe sites for these previously published FMDV assays. Nucleotides at positions 869, 8019, and 8028 of FMDV O_1_ Manisa (GenBank accession number AY593823) were substituted from A to C, C to T, and T to C, respectively, to reduce the nucleotide mismatch of the engineered construct with the primer and probe sequences used in the FMDV diagnostic assay. Thus, the FMDV surrogate RNA sequence with added ApaI and AvrII flanking sites was synthesised by GeneArt (Life Technologies). Following digestion with ApaI and AvrII, the sequence was inserted into similarly digested plasmid pBIN-mim-AvrII-mp del to yield pBIN-mim-AvrII-mp del-FMDV (Fig. 1). The transformation of plasmids into *Agrobacterium tumefaciens* strain LBA4404, the infiltration of cowpea (*Vigna unguiculata*) plants and the propagation and purification of virus particles was carried out as described by King et al. (2007).

### 2.3 Evaluation of the template encapsidated in the CPMV particles using molecular assays for the detection of FMDV

A sample matrix was first prepared by mixing a 10% (w/v) of negative bovine epithelium (bought from a local abattoir) with M25 phosphate buffered saline (35 mM Na_2_HPO_4_; 5.7 mM KH_2_PO_4_; pH 7.6) and then filtering the final solution. Then, a 10 fold dilution series was prepared using the recombinant CPMV (at 1 μg/μl) in the negative bovine epithelium suspension. This sample matrix was used since it most closely represented clinical material that might be received for diagnostic investigation. From each dilution, viral RNA was extracted (in triplicate) using the QIAmp Viral RNA Mini kit (Qiagen, UK) with an elution volume of 40 μl (according to manufacturer's instructions).

### 2.4 RNA dependent amplification using conventional RT-PCR

Initial experiments using conventional RT-PCR protocols were undertaken to confirm that the amplified template was RNA, and that residual DNA from the original plasmid construct did not influence the results from the molecular tests. In these experiments, nucleic acid prepared from the dilution series of CPMV was divided into two batches; one was subjected to amplification by RT-PCR (Reid et al., 2000; Rodriguez et al., 1994), while an experiment in parallel used identical conditions without the addition of the RT step. The following protocol was used: 5 μl of nucleic acid was added to 7.5 μl nuclease-free water and 0.5 μl random hexamers (500 μg/ml) (Promega, USA). This mix was denatured at 65 °C for 5 min after which the reaction mix was held on ice. To this reaction, 7 μl of a solution containing 4 μl of 5× first strand buffer (Invitrogen), 1 μl RNase out (40 U/μl, Invitrogen), 1 μl dNTPs (10 μM each) and 1 μl Superscript III Reverse Transcriptase (200 U/μl, Invitrogen) was added. RT reaction was performed using the following protocol: 50 °C for 1 h and 70 °C for 15 min. 5 μl cDNA sample was added to 40 μl PCR mix comprising 5 μl of 10X buffer (Invitrogen), 2 μl MgSO_4_ (50 mM, Invitrogen), 1 μl dNTPS (10 μM each), 1 μl of both 1F forward and 1R reverse primers (10 μM), 0.2 μl Platinum Taq (Invitrogen, UK) and 34.8 μl nuclease free water. Both batches of samples – with or without the RT enzyme in the incubation step – were amplified in parallel using the following protocol: One cycle of 94 °C for 5 min; 40 cycles of 94 °C for 1 min, 50 °C for 1 min, 72 °C for 1 min and one cycle of 72 °C for 7 min. Following this, 10 μl of the PCR amplification of 10^−2^ to 10^−9^ dilution series was mixed with 2 μl loading buffer (Fermentas, Germany). Samples were then loaded in 1% (w/v) agarose and run at 100 V for 40 min.

### 2.5 Detection by diagnostic real-time RT-PCR assays

The template encapsidated in the CPMV particles was evaluated using two diagnostic RT-qPCR assays (Reid et al., 2002; Callahan et al., 2002) using a one-step RT-qPCR protocol previously described (Reid et al., 2009). Briefly, 5 μl of 10^−2^–10^−9^ dilution RNA samples prepared previously was added to 20 μl reaction mix encompassing 12.5 μl of 2× mix (Invitrogen); 2 μl forward primer (10 μM); 2 μl reverse primer (10 μM); 1.5 μl probe (5 μM); 1.5 μl nuclease free water and 0.5 μl SuperScript III/Taq Polymerase (Invitrogen, UK). One step RT-qPCR was performed using the following protocol: one cycle at 60 °C for 30 min; one cycle at 95 °C for 10 min and 50 cycles at 95 °C for 15 s and 60 °C for 1 min. *C*_*T*_ (cycle threshold) values were assigned as previously described (Reid et al., 2002).

### 2.6 Detection by LAMP assays

The performance of the template encapsidated in the CPMV particles was also evaluated in FMDV-specific RT-loop-mediated isothermal amplification (RT-LAMP) assays (Dukes et al., 2006; Shao et al., 2010). In these experiments, 5 μl of the 10^−2^ dilution RNA sample was added to 20 μl reaction mix containing 2.5 μl of 10× Thermopol buffer (Invitrogen), 1 μl of a 50 μM stock of internal primers (IPs: BIP and FIP), 1 μl of a 5 μM stock of external primers (EPs: F3 and B3), 1 μl of a stock of 25 μM of loop primers (Floop and Bloop), 0.5 μl of 10 mM of dNTPs, 0.5 μl of 100 mM of MgSO_4_, 5 μl of 5 M of betaine (Sigma Aldrich), 5 μl of dye mix, 1.3 μl of nuclease free water, 2 μl of 8 U/μl of *Bst* polymerase (Invitrogen) and 0.2 μl of 15 U/μl of Cloned AMV RT (Invitrogen) to a final volume of 25 μl. RT-LAMP was carried out on the Mx3005P machine (Stratagene) under the following conditions: 65 °C for 60 min and 85 °C for 5 min. After detection on the Mx3005P machine, amplified products were visualised using agarose gel electrophoresis.

### 2.7 Thermo-stability of the recombinant CPMV particle

Long term stability of CPMV particles was investigated over a 46 days period. Two hundred and seventy six aliquots were prepared using a 10^−3^ CPMV dilution (∼1 ng/μl) sample in 10% negative bovine epithelium suspension. Aliquots were divided into two batches; one was stored at room temperature conditions (20–26 °C) while the other was held at 37 °C. After incubation for set of periods of time, triplicate aliquots were stored in RLT lysis buffer (Qiagen, Germany) and frozen at −80 °C until they were analysed. Once the experiment was finished, nucleic acid was extracted from all samples using the QIAmp Viral RNA Mini kit (Qiagen, Germany) according to the manufacturer's protocol with 40 μl elution volume. Then, 5 μl RNA sample was added to 20 μl RT-qPCR master mix as described above and a one step RT-qPCR reaction targeting the FMDV 3D region was performed.

## 3 Results

### 3.1 Production of CPMV particles based on movement deficient virus

**Fig. 3:**
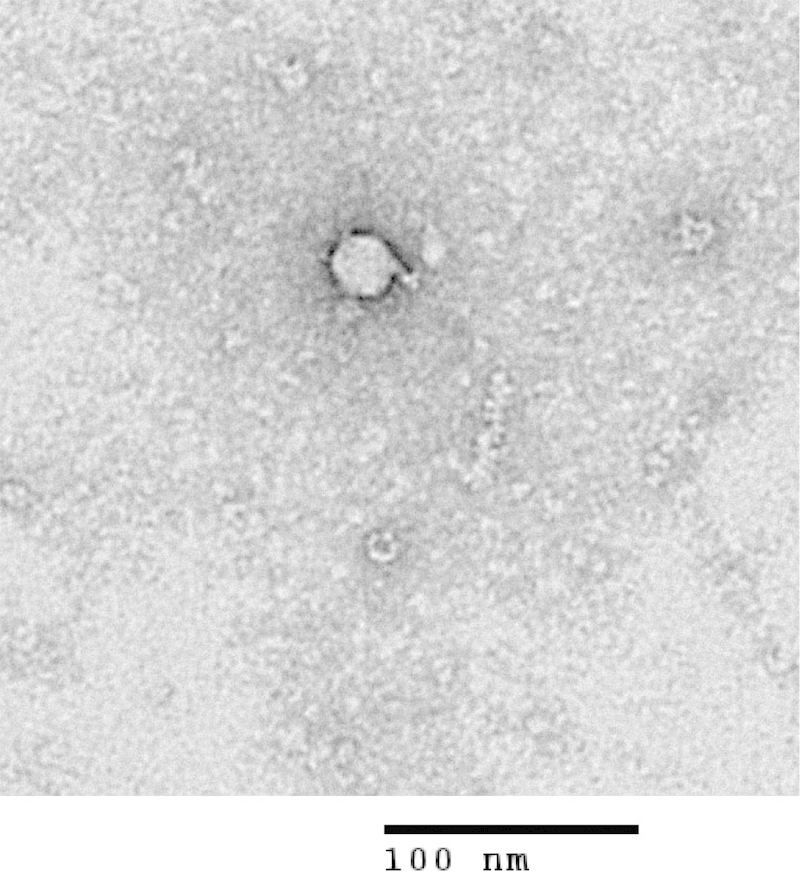
Recombinant Cowpea Mosaic Virus particle containing surrogate FMDV RNA sequences visualised by electron microscope.

Agroinfiltration of ten cowpea plants with pBIN-mim-AvrII-mp del or pBIN-mim-AvrII-mp del-FMDV, containing the deleted version of the 48K movement protein, did not result in disease associated symptoms appearing on the upper leaves, suggesting that any infection was restricted to the area which was infiltrated. Sap extracted from the inoculated tissue was unable to initiate an infection on healthy cowpeas; by contrast sap extracted from leaves of five cowpea plants infiltrated with constructs with an intact 48K protein gave 100% infection when passaged to healthy plants. These results confirmed that the deletion within the 48K protein had effectively abolished the infectivity of the virus. Purification of particles from infiltrated tissue gave yields of 3–4 mg per kg wet weight leaf tissue, approximately 1% of the yield obtained with wild-type virus. This is consistent with the inability of the virus to move out of the initially infected cells. Transmission electron microscopy revealed that the preparations contained particles indistinguishable from wild-type CPMV (Fig. 3).

### 3.2 RNA dependent amplification of the template encapsidated within the CPMV particles

**Fig. 4:**
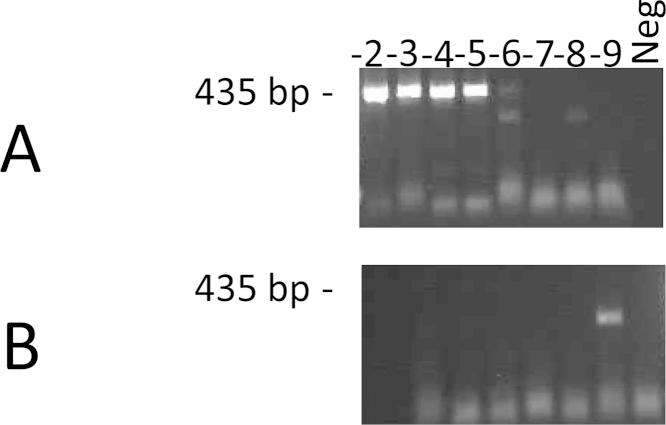
Conventional PCR detection of the FMDV surrogate RNA template with (A) or without (B) reverse transcription step. Figure shows an agarose-gel visualisation of the Log dilutions of CPMV particles 10^−2^ (−2); 10^−3^ (−3); 10^−4^ (−4); 10^−5^ (−5) 10^−6^ (−6), 10^−7^ (−7) 10^−8^ (−8) 10^−9^ (−9) and negative bovine epithelium suspension (Neg) using PCR primers (1F/1R) that target the 5′UTR of the FMDV genome.

The nature of the template contained in the engineered CPMV positive controls was tested using nucleic acid extracted from a dilution series of the CPMV particles. Dilutions (10^−2^ to 10^−5^: wells 1–4 of Fig. 4A) which were subjected to a RT step prior to PCR amplification generated an expected amplification band around 435 bp on the agarose gel. However, parallel samples which were only amplified by PCR without a RT step displayed no amplification bands (wells 1–4 of Fig. 4B). Moreover, nucleic acid extracted from negative bovine suspension sample failed to exhibit an amplification signal on the agarose gel in either condition (well 9 of Fig. 4A and B). These results confirmed that the positive signal generated from the recombinant CPMV particles was RNA-dependent.

### 3.3 Detection of the template encapsidated in the CPMV particles using previously published diagnostics assays

**Fig. 5:**
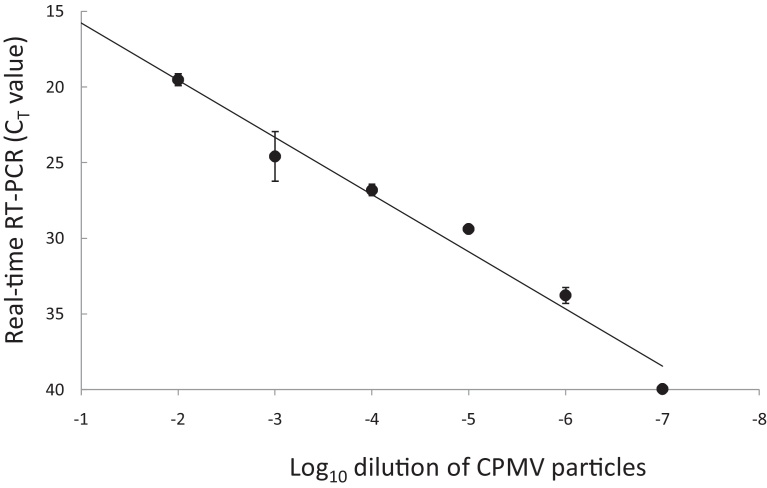
Detection of FMDV surrogate viral RNA in the recombinant CPMV particle using the RT-qPCR targeting FMDV 3D. Points shown represent mean *C_T_* ± SD of triplicate determinations of a 10-fold dilution series of the CPMV particle (starting at 1 μg/μl) in negative bovine epithelium suspension.

**Fig. 6:**
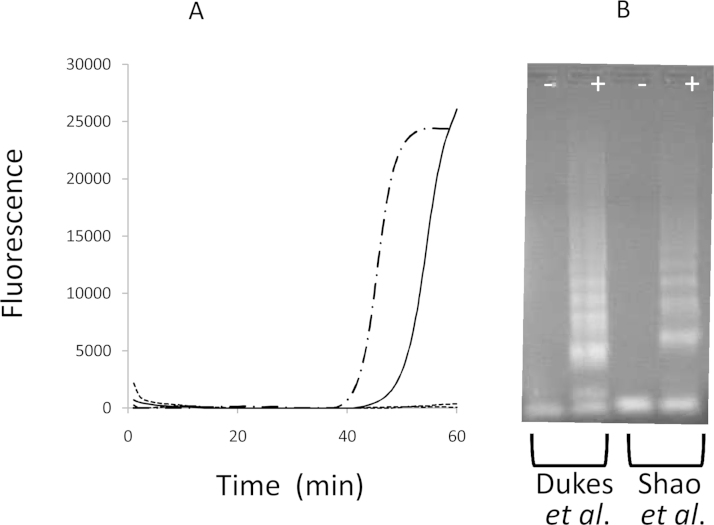
Use of RT-LAMP to detect the FMDV surrogate RNA encapsidated in the recombinant CPMV particles. Real-time amplification curves (A) are shown for two RT-LAMP assays (Dukes et al., 2006: [dashed line] and Shao et al., 2010 [continuous line]) using a CPMV RNA positive sample. Results for negative bovine epithelial suspension sample did not generate an amplification signal (corresponding curves shown at the bottom of the figure). [B] Post-amplification analysis of these positive (+) and negative (−) samples by agarose-gel electrophoresis is shown for the two RT-LAMP assays.

A decimal titration series from 10^−2^ to 10^−9^ prepared with recombinant CPMV particles in 10% negative bovine epithelium suspension has been tested for previously published diagnostic assays. Results confirmed the ability of the nucleic acid extracted from the dilution series to be positively amplified using the FMDV RT-qPCR 3D assay (Fig. 5). Similar results were generated using RT-qPCR targeting the FMDV 5′UTR region (results not shown). One representative sample from the dilution series was used to test two RT-LAMP assays (Dukes et al., 2006 and Shao et al., 2010). Fig. 6A demonstrates positive amplification with RNA at around 40 min and 43 min for the Dukes et al. (2006), and Shao et al. (2010), RT-LAMP assays, respectively. Nucleic acid extracted from negative bovine controls failed to generate a positive curve using either RT-LAMP assays. These results generated using a real-time PCR machine were confirmed using agarose gel electrophoresis which revealed laddering patterns characteristic of post amplification LAMP products (Fig. 6B).

### 3.4 Thermo-stability of the recombinant CPMV particles

**Fig. 7:**
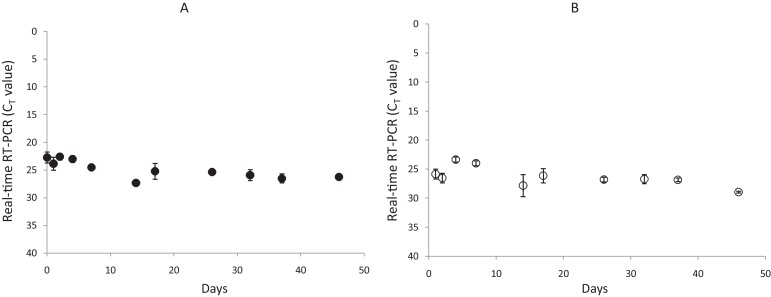
Stability of the recombinant CPMV particles at room temperature (range 20–26 °C (A)) and at 37 °C (B). *C_T_* values shown (mean ± SD) of independent triplicates) were determined using the FMDV 3D RT-qPCR assay.

Long term thermo-stability of recombinant CPMV particles has been monitored over a period of 46 days. All RNA samples extracted from the recombinant CPMV particles previously stored at room temperature (20–25 °C) or stored at 37 °C generated positive amplification throughout the 46 days period, displaying *C*_*T*_ values ranging from 23 to 27 (Fig. 7).

## 4 Discussion

FMD is a highly infectious disease of both domestic and wild cloven-hoofed animals. FMDV (Family: Picornaviridae, genus: *Aphthovirus*), the causative agent is normally handled in high-containment laboratories that employ robust bio-security measures to prevent exposure to susceptible animals. Conventional approaches used to prepare positive controls and standard materials that can be shipped between laboratories rely on the use of inactivated material containing infectious FMDV. The use of these materials for proficiency testing schemes (Ferris et al., 2006), particularly in those laboratories that work at lower containment levels requires that validated inactivation approaches are used. However, the chemical (BEI) treatment to inactivate the live virus requires very expensive and time consuming in vitro and in vivo testing in order to verify the non-infectivity of the final product.

In this study, a surrogate FMDV RNA was engineered and encapsidated into a CPMV particle. This RNA contains a 512 nucleotide RNA fragment comprising the sequence targets for six pan-serotype assays used for routine diagnostics of FMD: two RT-qPCRs, targeting either the 5′UTR (Reid et al., 2002) or 3D encoding regions (Callahan et al., 2002), two conventional RT PCRs (Reid et al., 2000; Rodriguez et al., 1994), an RT linear-after-the exponential PCR (Pierce et al., 2010; Reid et al., 2010) and two RT-LAMP assays (Dukes et al., 2006; Shao et al., 2010). These CPMV particles are safe, since they do not contain coherent sequences that can revert to infectious FMD virus. Furthermore, the recombinant CPMV used to generate the particles was engineered so that it is unable to move from cell to cell and is thus unable to cause a productive infection of the plants. This was achieved by generating an in-frame deletion within the 48K region of RNA-2-encoded polyprotein. Such a deletion has previously been shown to allow particle formation in individual plant cells but to abolish the ability of the virus to cause a productive infection in whole plants (Wellink and van Kammen, 1989). The deletion reduced the yield of particles produced in inoculated leaves to approximately 1% of the yield obtained with the wild type virus. However, this lower yield still gives sufficient particles for millions of PCR-based diagnostic reactions. Thus, this system represents a bio-safe option with no risk of accidentally spreading FMDV in the environment. Alternative approaches used to generate encapsidated or armoured RNA exploit MS2 bacteriophage coat protein which provides a protective shell for the RNA, enabling storage at 4 °C for 60 days (Pasloske et al., 1998). These recombinant CPMV-like particles offer a more robust protection for the RNA and its surrounding environment since the CPMV-based particles can survive for a period of 46 days at 37 °C without any loss of the RNA when detected using RT-qPCR (Fig. 7). Furthermore, CPMV particles can survive at 60 °C for at least one hour, across the range of pH 4–9 (Montague et al., 2011). Therefore, our surrogate FMDV RNA packaged in the CPMV particle could easily be shipped at room temperature and could also be used in countries where maintaining the cold chain could be a challenge.

In addition to their use as standards and positive controls in National Reference Laboratories (NRL's), these recombinant CPMV particles have wider applications in other diagnostic scenarios. In particular, they can be used to prepare biosafe surrogate spiked materials that could be used to evaluate molecular diagnostic assays and kits outside of government high-containment facilities, and as positive controls for devolved or pen-side tests that are now under development for FMDV detection in disease-free and endemic settings (Madi et al., 2012; King et al., 2008). Furthermore, although this study has focussed on FMDV, this is a generic approach that can be easily applied to many other RNA viruses that infect animals, plants and man.

## Figures and Tables

**Table 1:** Sequences of primer recognition sites present in the FMDV positive control.

Primer name	Sequence (5′ to 3′)	Genomic location^a^	Position in construct	Reference
1F forward	GCCTGGTCTTTCCAGGTCT	680–698	21–39	Reid et al. (2000)
1F reverse	CCAGTCCCCTTCTCAGATC	1007–989	493–475	

IRES Forward	CACYTYAAGRTGACAYTGRTACTGGTAC	898–925	78–105	Reid et al. (2002)
IRES Reverse	CAGATYCCRAGTGWCICITGTTA	994–972	156–134	
IRES probe	CCTCGGGGTACCTGAAGGGCATCC	971–948	151–128	

Rodriguez 3D forward	CAGAGATGTGGAAGAGCGCG	6702–6721	1–20	
Rodriguez 3D reverse	GCGGAACAGCGCTTTGTCC	6909–6891	512–494	

Callahan 3D forward	ACTGGGTTTTACAAACCTGTGA	7870–7891	250–271	Callahan et al. (2002)
Callahan 3D reverse	GCGAGTCCTGCCACGGA	7976–7960	356–340	
Callahan 3D probe	TCCTTTGCACGCCGTGGGAC	7921–7940	301–320	

CHN-F3	TGTGATGGCTTCGAAGACC	7887–7905	267–285	Shao et al. (2010)
CHN-B3	TGCGTCACCGCACACG	8094–8079	474–459	
CHN-FIP	TGCCACGGAGATCAACTTCTCCTTTTCTCGAGGCTATCCTCTCCTT	7968–7923	348–303	
CHN-BIP	GAGTACCGGCGTCTCTTTGAGCTTTTCGTTCACCCAACGCAGGTAA	7996–8041	376–421	
CHN-LF	TGTATGGTCCCACGGCG	7946–7930	326–310	
CHN-LB	TTGAGCCTTTCCAGGGCC	8012–8029	392–409	

UK-F3	CATGGACTATGGAACTGGGT	7857–7876	237–256	Dukes et al. (2006)
UK-B3	GGCCCTGGAAAGGCTCA	8019–8003	409–393	
UK-FIP	CACGGCGTGCAAAGGAGAGGATTTTACAAACCTGTGATGGCTTCG	7855–7899	235–279	
UK-BIP	GGAGAAGTTGATCTCCGTGGCATTTTAAGAGACGCCGGTACTCG	7947–7990	327–370	
UK-LF	TAGCCTCGAGGGTCTTCG	7892–7909	295–278	
UK-LB	GGACTCGCCGTCCA TCT	7969–7985	267–285	

a Primer locations correspond to the complete genome sequence of the FMDV reference virus O_1_ Manisa (GenBank accession number AY593823).

## References

[bib0010] Callahan J.D., Brown F., Osorio F.A., Sur J.H., Kramer E., Long G.W., Lubroth J., Ellis S.J., Shoulars K.S., Gaffney K.L., Rock D.L., Nelson W.M. (2002). Use of a portable real-time reverse transcriptase-polymerase chain reaction assay for rapid detection of foot-and-mouth disease virus. J. Am. Vet. Med. Assoc..

[bib0015] Dukes J.P., King D.P., Alexandersen S. (2006). Novel reverse transcription loop-mediated isothermal amplification for rapid detection of foot-and-mouth disease virus. Arch. Virol..

[bib0020] Ferris N.P., King D.P., Reid S.M., Hutchings G.H., Shaw A.E., Paton D.J., Goris N., Hass B., Hoffmann B., Brocchi E., Bugnetti M., Dekker A., De Clerq K. (2006). Foot-and-mouth disease virus: a first inter-laboratory comparison trial to evaluate virus isolation and RT-PCR detection methods. Vet. Microbiol..

[bib0025] Gopinath K., Wellink J., Porta C., Taylor K.M., Lomonossoff G.P., van Kammen A. (2000). Engineering cowpea mosaic virus RNA-2 into a vector to express heterologous proteins in plant. Virology.

[bib0030] Heath G.S., King D.P., Turner J.L., Wakeley P.R., Banks M. (2003). Use of an internal standard in a TaqMan nested reverse transcription chain reaction for the detection of bovine diarrhoea virus. Vet. Microbiol..

[bib0035] Hoffmann B., Beer M., Reid S.M., Mertens P., Oura C.A., van Rijn P.A., Slomka M.J., Banks J., Brown I.H., Alexander D.J., King D.P. (2009). A review of RT-PCR technologies used in veterinary virology and disease control: sensitive and specific diagnosis of five livestock diseases notifiable to the World Organisation for Animal Health. Vet. Microbiol..

[bib0040] Hoffmann B., Depner K., Schirrmeier H., Beer M. (2006). A universal heterologous internal control system for duplex real-time RT-PCR assays used in a detection system for pestiviruses. J. Virol. Methods.

[bib0045] Liu L., Lomonosoff G.P. (2002). Agroinfection as a rapid method for propagating cowpea mosaic virus-based constructs. J. Virol. Methods.

[bib0050] King D.P., Montague N., Ebert K., Dukes J.P., Schädlich L., Belsham G.J., Lomonossoff G.P. (2007). Development of a novel recombinant encapsidated RNA particle: evaluation as an internal control for diagnostic RT-PCR. J. Virol. Methods.

[bib0055] King D.P., Dukes J.P., Reid S.M., Ebert K., Shaw A.E., Mills C.E., Boswell L., Ferris N.P. (2008). Prospects for rapid diagnosis of foot-mouth disease in the field using reverse transcriptase-PCR. Vet. Rec..

[bib0060] Madi M., Hamilton A., Squirrell D., Mioulet V., Evans P., Lee M., King D.P. (2012). Rapid detection of foot-and-mouth disease virus using a field-portable nucleic acid extraction and real-time PCR amplification platform. Vet. J..

[bib0065] Monger W., Alamillo J.M., Sola I., Perrin Y., Bestagno M., Burrone O.R., Sabella P., Plana-Duran J., Enjuanes L., Garcia J.A., Lomonossoff G.P. (2006). An antibody derivative expressed from viral vectors passively immunizes pigs against transmissible gastroenteritis virus infection when supplied orally in crude plants extracts. Plant Biotechnol. J..

[bib0070] Montague N.P., Thuenemann E.C., Saxena P., Saunders K., Lenzi P., Lomonossoff G.P. (2011). Recent advances of cowpea mosaic virus-based particle technology. Hum. Vaccines.

[bib0125] Pasloske B.L., Walkerpeach C.R., Oberwoeller R.D., Winkler M., DuBois D.B. (1998). Armored RNA technology for production of ribonuclease-resistant viral RNA controls and standards. J. Clin. Microbiol..

[bib0075] Pierce K.E., Mistry R., Reid S.M., Bharya S., Dukes J.P., Hartshorn C., King D.P., Wangh L.J. (2010). Design and optimization of a novel reverse transcription linear-after-the exponential PCR for the detection of foot-and-mouth disease virus. J. Appl. Microbiol..

[bib0080] Reid S.M., Ferris N.P., Hutchings G.H., Samuel A.R., Knowles N.J. (2000). Primary diagnosis of foot-and-mouth disease by reverse transcription polymerase chain reaction. J. Virol. Methods.

[bib0085] Reid S.M., Ferris N.P., Hutchings G.H., Zhang Z., Belsham G.J., Alexandersen S. (2002). Detection of all seven serotypes of foot-and-mouth disease virus by real time, fluorogenic reverse-transcription polymerase chain reaction assay. J. Virol. Methods.

[bib0090] Reid S.M., Ebert K., Bachanek-Bankowska K., Batten C., Sanders A., Wright C., Shaw A.E., Ryan E.D., Hutchings G.H., Ferris N.P., Paton D.J., King D.P. (2009). Performance of real-time RT-PCR for the detection of foot-and-mouth disease virus during field outbreaks in the United Kingdom in 2007. J. Vet. Diagn. Invest..

[bib0095] Reid S.M., Pierce K.E., Mistry R., Bharya S., Dukes J.P., Volpe C., Wangh L.J., King D.P. (2010). Pan-serotypic detection of foot-and-mouth disease virus by RT-linear-after-the-exponential PCR. Mol. Cell. Prob..

[bib0100] Rodriguez A., Nủñez J.I., Nolasco G., Ponz F., Sobrino F., de Blas C. (1994). Direct PCR detection of foot-and-mouth disease virus. J. Virol. Methods.

[bib0105] Sainsbury F., Lavoie P-O., D’Aoust M-A., Vezina L-P., Lomonossoff G.P. (2008). Expression of multiple proteins using full-length and deleted versions of Cowpea Mosaic virus RNA-2. Plant Biotechnol. J..

[bib0110] Shao J.J., Chang H.Y., Zhou G.Q., Cong G.Z., Du J.Z., Lin T., Gao S.D., He J.J., Liu X.T., Liu J.X., Gao J.L. (2010). Rapid detection of foot-and-mouth disease virus by reverse transcription loop-mediated isothermal amplification (RT-LAMP). Int. J. Appl. Res. Vet. Med..

[bib0115] Wellink J., van Kammen A.B. (1989). Cell-to-cell transport of cowpea mosaic virus requires both the 58K/48K proteins and the capsid proteins. J. Gen. Virol..

[bib0120] Westcott D.G., King D.P., Drew T.W., Nowotny N., Kindermann J., Hannant D., Belák S., Paton D.J. (2003). Use of an internal standard in a closed one-tube RT-PCR for the detection of equine arteritis virus RNA with fluorescent probes. Vet. Res..

